# Homogenous 96-Plex PEA Immunoassay Exhibiting High Sensitivity, Specificity, and Excellent Scalability

**DOI:** 10.1371/journal.pone.0095192

**Published:** 2014-04-22

**Authors:** Erika Assarsson, Martin Lundberg, Göran Holmquist, Johan Björkesten, Stine Bucht Thorsen, Daniel Ekman, Anna Eriksson, Emma Rennel Dickens, Sandra Ohlsson, Gabriella Edfeldt, Ann-Catrin Andersson, Patrik Lindstedt, Jan Stenvang, Mats Gullberg, Simon Fredriksson

**Affiliations:** 1 Olink Bioscience, Uppsala, Sweden; 2 Section for Molecular Disease Biology and Sino-Danish Breast Cancer Research Centre, Department of Veterinary Disease Biology, Faculty of Health and Medical Sciences, University of Copenhagen, Copenhagen, Denmark; Deutsches Krebsforschungszentrum, Germany

## Abstract

Medical research is developing an ever greater need for comprehensive high-quality data generation to realize the promises of personalized health care based on molecular biomarkers. The nucleic acid proximity-based methods proximity ligation and proximity extension assays have, with their dual reporters, shown potential to relieve the shortcomings of antibodies and their inherent cross-reactivity in multiplex protein quantification applications. The aim of the present study was to develop a robust 96-plex immunoassay based on the proximity extension assay (PEA) for improved high throughput detection of protein biomarkers. This was enabled by: (1) a modified design leading to a reduced number of pipetting steps compared to the existing PEA protocol, as well as improved intra-assay precision; (2) a new enzymatic system that uses a hyper-thermostabile enzyme, Pwo, for uniting the two probes allowing for room temperature addition of all reagents and improved the sensitivity; (3) introduction of an inter-plate control and a new normalization procedure leading to improved inter-assay precision (reproducibility). The multiplex proximity extension assay was found to perform well in complex samples, such as serum and plasma, and also in xenografted mice and resuspended dried blood spots, consuming only 1 µL sample per test. All-in-all, the development of the current multiplex technique is a step toward robust high throughput protein marker discovery and research.

## Introduction

There is a continuous need for new, and better, blood-based biomarkers to develop minimally invasive tools for screening and diagnostic purposes. Several studies have also demonstrated that higher power of discrimination can be obtained by combining more than one biomarker [1], [2], [3], [4]. This demands the use of multiplex techniques for high-throughput. Several immunoassays, such as bead-based and planar arrays, used to quantify multiple proteins are currently available. These methods require extensive optimizations to eliminate antibody cross-reactivity [5]. This drawback increases the development time for new assays and limits the multiplexing level to around ten due to the exponential increase in possible cross-reactive events with a higher degree of multiplexing.

The proximity ligation assay (PLA) and, more recently, the proximity extension assay (PEA) have been developed as homogenous immunoassays shown to be both sensitive and specific [6], [7], [8]. PEA is based on pairs of antibodies that are linked to oligonucleotides having slight affinity to one another (PEA probes). Upon target binding the probes are brought in proximity, and the two oligonucleotides are extended by a DNA polymerase forming a new sequence that now acts as a unique surrogate marker for the specific antigen [6], [7]. This sequence is typically quantified by quantitative real-time PCR (qPCR), where the number of PCR templates formed is proportional to the initial concentration of antigen in the sample.

Immunoassay robustness and precision are crucial for implementation in research and routine diagnostics. By virtue of the proximity requirement for template formation and the stringency attained from the qPCR readout, antibody cross-reactivity is unlikely to be detected and cause problems in PEA. Therefore we postulated that PEA would be more scalable compared to traditional immunoassays [9]. In the present paper we present the development of a 96-plex PEA-based immunoassay and report its performance upon the simultaneous measurement of 92 known or putative protein biomarkers for cancer. Multiplexing did not bring about any unspecific antibody binding events or any other type of assay interference and, thus, both a high assay sensitivity and specificity was maintained throughout the assay panel. With the current data we put forward the 96-plex PEA immunoassay as an approach to facilitate a systematic and flexible exploration of protein profiles in body fluids, such as serum or plasma. We have also successfully performed multiplex PEA analyses of dried blood spots and sera from xenografted mice, two applications where low sample consumption is critical.

## Materials and Methods

### Antigens, Antibodies, DNA Polymerases, and other Reagents

Recombinant antigens were resuspended to 100 µg/mL in PBS with 0.1% BSA and pooled either in sub mixes of 10 or 20, or in a mix containing all of the antigens with the exception of CA125 and CA242 that were excluded due their origin in human cells/tissue and the resulting risk of contaminating proteins (Table S1). Antibodies, either matched monoclonal antibodies (mAb) or a polyclonal antibody (pAb) batch split in two, were resuspended at 1 mg/mL in PBS. All antibodies were purchased from commercial sources. For the IL-6 assays used for Figure S1 and Table S2, the following antibody (Ab) combinations were used: A mAb/B mAb: A = no. MAB206, clone 6708 (RnD Systems), B = no. 554541, clone MQ2-13A5 (BD Pharmingen); A mAb/B pAb: A = no. MAB206, clone 6708, B = no. AF-206-NA (RnD Systems); A pAb/B mAb: A = no. AF-206-NA, B = no. 554541, clone MQ2-13A5; A pAb/B pAb: A/B = no. AF-206-NA.

The hyper-thermostable DNA polymerases; Pwo Hypernova (DNA Gdansk), Pfu (Thermo Fisher Scientific Inc., Waltham, MA), TLA (Bioneer, Daejeon, South Korea), Pwo Delta3 (DNA Gdansk), KOD exo+, KOD exo− (Toyobo, Osaka, Japan), and DreamTaq (Thermo Fisher Scientific Inc) were evaluated at 0.5 units per reaction with the following thermocycling protocol: 10 min room temperature (RT) incubation, 23 min extension at RT/37°C/45°C, 10 min denaturation at 85°C.

Dilution series of bilirubin (Sigma-Aldrich) and intra-lipid (Fresenius Kabi, Uppsala Sweden) were prepared by making two-fold dilutions in H_2_O ranging from 200–6300 µg/mL and 3–200 mg/mL, respectively. When added to human serum samples the final concentrations were between 20–630 µg/mL bilirubin and 0.3–20 mg/mL intralipid. Hemolysate samples were prepared at final concentrations ranging from 0.23–15 g/L, where 15 g/L corresponds to complete lysis of 10% of all blood cells in a sample. Two whole heparinized blood samples (27 mL at Hb 159 g/L) were spun in 50 ml Falcon tubes at 3000 rpm, 10 min at 10°C, without brake. Cell pellets were washed four times with 0.9% NaCl, resuspended in H_2_O to the original volume, freeze-thawed, vigorously mixed four times, pooled, and spun 5000 rpm, 20 min at 10°C. The final Hb-value in the hemolysate stock solution was estimated to 150 g/L.

Human serum samples were purchased from 3H Biological (Uppsala, Sweden).

### Generation of PEA Probes

**Figure 1:**
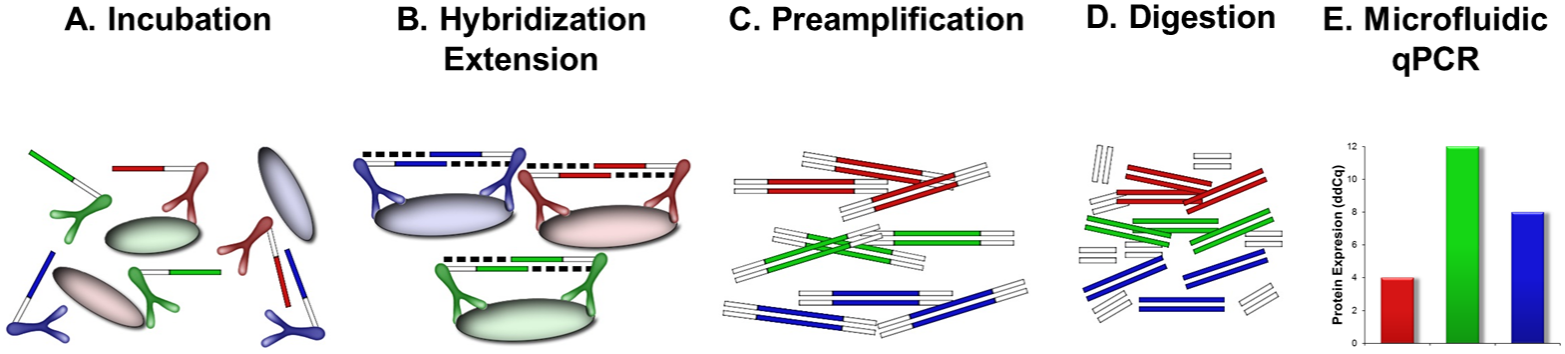
Design and description of 96-plex PEA. (A) 94 pairs of specific antibodies are equipped with oligonucletotides (PEA probes) and mixed with an antigen-containinig sample. (B) Upon sample incubation, all proximity probe pairs bind their specific antigens, which brings the probe oligonucleotides in close proximity to hybridize. The oligonucleotides have unique annealing sites that allows pair-wise binding of matching probes. Addition of a DNA polymerase leads to an extension and joining of the two oligonucleotides and formation of a PCR template. (C) Universal primers are utilized to preamplify all 96 different DNA templates in paralell. (D) Uracil-DNA glycosylase partly digests the DNA templates and remove all unbound primers. (E) Finally each individual DNA sequence is detected and quantified using specific primers in by microfluidic qPCR.

PEA probes were generated using succinimidyl-4-(N-maleimidomethyl)cyclohexane-1-caroxylate (SMCC)-driven coupling of two paired antibodies (specific for each target antigen) to unique oligonucleotides (A and B) each comprising binding sites for universal amplification primers and specific detection primers, one site for pair-wise annealing between oligonucleotide A and B, and one universal site for binding of molecular beacon and detection in qPCR (Figure 1). The performance of all specific qPCR primers has been evaluated by determining their amplification efficiencies. Primers displaying <80% efficiency were replaced.

Antibodies, either matched monoclonal antibodies (mAb) or a polyclonal antibody (pAb) batch split in two, were resuspended at 1 mg/mL in PBS. A 40 kDa Zeba plate (Thermo Scientific, Rockford, IL) was equilibrated four times with 100 mM phosphate buffer, pH 7.3 (250 µL/well,1000×g, 2 min). 20 µL antibody was added/well, spun 1000×g, 3 min, and collected in a new plate. Sulfo-SMCC (Thermo Scientific) was dissolved to 3.33 mM in 100 mM phosphate buffer. 2 µL sulfo-SMCC was added to each antibody well, mixed, and incubated at 4°C for 2 h with three times intermittent mixing. A new 40 kDa Zeba plate was equilibrated with 100 mM phosphate buffer with 20 mM EDTA. The SMCC-treated antibodies were transferred to the Zeba plate and spun at 1000×g, 3 min. Conjugation protocols were previously optimized to function well on both mAb and pAb.

Oligonucleotides synthesized with a 5′-thiol modification (Integrated DNA Technologies, Leuven, Belgium) were resuspended at 1 mM in 100 mM phosphate buffer with 20 mM EDTA, and distributed in a 96-well plate in 1.3 µL. 2.2 µL 40 mM DTT (Life Technologies, Eugene, OR) was added to 1.3 µL of each oligonucleotide, mixed, and incubated at 95°C, 2 min, followed by a 1 h incubation step at 37°C. 20 µL PBS buffer with 20 mM EDTA was added to the oligonucleotides and excess DTT was removed using two consecutive 7 kDa Zeba plates equilibrated with 100 mM phosphate buffer.

The SMCC-treated antibodies were mixed with the DTT-treated oligonucleotides at 10x molar excess of oligo to Ab and transferred to pre-wet Slide-A-Lyzer Mini 7 MWCO dialysis cups (Thermo Scientific) and dialyzed in 1 L PBS with 5 mM EDTA at 4°C for two days with one buffer exchange to PBS. The PEA probes were finally diluted to 75 µg/mL in a PBS buffer containing 4 mM EDTA, 35 µg/mL ssDNA (Sigma-Aldrich), 0.1% fish gelatin, and 20 mM Tris HCl, and stored at 4°C.

### Proximity Extension Analysis

One µL sample (buffer (PBS with 0.1% BSA), antigen-spiked buffer, or biological sample) was mixed with 0.3 µL of each proximity probe mix (A and B), 0.3 µL Incubation Stabilizer (Olink Bioscience, Uppsala, Sweden) and 2.1 µL Incubation Solution (Olink Bioscience) and incubated overnight at 4°C (Figure 1A). A combined extension and preamplification mix (96 µL) containing 10 µL MUX PEA Solution (Olink Bioscience), 0.5 units Pwo (DNA Gdansk, Poland), 1 µM forward + reverse universal preamplification primers, and 1 unit hot-start DNA polymerase was added to each reaction at RT. After mixing and a total 5-min incubation, the plate was transferred to a thermocycler (Applied Biosystem 2720) running an initial extension step to unite the two oligonucleotides (50°C, 20 min), immediately followed by a hot-start step (95°C, 5 min) and 17 cycles of amplification (95°C, 30 s; 54°C, 1 min; 60°C, 1 min) (Figure 1B). Amplification was performed with universal flanking primers to amplify all 96 sequences in parallel (Figure 1C). Finally, 2.8 µL of the preamplification products were mixed with 7.2 µL buffer containing 5 µL MUX Detection Solution (Olink Bioscience), 0.071 units Uracil-DNA glycosylase (DNA Gdansk) used to digest the DNA templates and remaining universal primers (Figure 1D), and 0.14 units hot-start polymerase. Five µL from the sample mix above was transferred to the sample inlet wells of a microfluidic real-time PCR chip (96.96 Dynamic Array IFC, Fluidigm Biomark). Five µL from respective well of an Assay Plate (Olink Bioscience) containing 9 µM sequence-specific internal detection primers, 2.5 µM molecular beacon in 1x DA Assay Loading Reagent (Fluidigm) were transferred to the assay inlet wells (Figure 1E). The chip was run in a Biomark instrument with the following program: Thermal mix (50°C, 2 min; 70°C, 30 min; 25°C; 10 min), Hot-start (95°C, 5 min), PCR Cycle 40 cycles (95°C, 15 s; 60°C, 1 min) according to the manufacturer’s guidelines (http://www.fluidigm.com/biomark-hd-system.html).

### Internal Spike-in Controls

Internal spike-in controls included in the incubation buffer (incubation controls green fluorescent protein (GFP) and phycoerythrin (PE), extension control, and detection control) were individually titrated to reach threshold cycle (Cq)-values between 12 and 15 (for robust measurements) and pooled. Resulting concentrations during incubation were 16, 1, 0.235, and 0.077 pM for GFP, PE, extension control, and detection control, respectively. For evaluation, all samples analyzed on a single chip were normalized against each of the different controls (linear value sample/linear value normalizer), after which the % coefficient of variation (CV) was calculated for the remaining three controls across all samples. The extension control was chosen as the best normalizer. For the Inter-Plate Control (IPC), concentrations were between 1 fM and 100 fM for the individual controls during incubation.

### Data Analysis and Normalization

For each data point, the raw Cq-value (log_2_ scale) was normalized by subtracting the Cq-value for the extension control reaction for the corresponding sample, thereby correcting for technical variation. The resulting delta Cq (dCq)-value was compared to that of the corresponding background reaction resulting in a ddCq-value, and used for most data presented herein, either in log_2_ scale, or as linearized values (2^ddCq^). For the interference data an additional data transformation step was included where the average ddCq value for a sample was divided with the value for 3 standard deviations (SD) of the corresponding replicate (in the figure denoted as relative signal). This step is performed simply for comprehensibility of the figure as it brings the level for ±3 SD for each assay to ±1.

For LOD/LLOQ/ULOQ calculations, a 4-Parameters non-Linear Regression analysis was performed on normalized protein expression (NPX) values (background values and all values beyond the plateau of the assay removed) using an algorithm of the FourParameterLogisticCurve class in the Extreme Numerics library (Extremeoptimization.com). Limit of detection (LOD) was defined as 3×SD above background. Lower limit of quantification (LLOQ) and upper limit of quantification (ULOQ) were defined as lowest and highest concentrations measured with %CV<30 and relative error (RE) <30%.

### Xenograft Experiments

Three-to-four-week-old female NMRI-nude mice (Taconic, Denmark) were housed in individually ventilated cages at a research animal facility at Uppsala University (Uppsala, Sweden) according to regulations. Human neuroblastoma cells (SK-N-FI) were mixed at a 1∶1 volume ratio with Matrigel (BD Biosciences, San Diego, CA) and 10^6^ cells were injected subcutaneously in the right-hind flank in 100 µL. Before and 30 days after tumor inoculation, 100–200 µL blood was collected from the tail vein and serum was extracted using Microvette Capillary Blood Collection System (Sarstedt, Numbrecht, Germany) and stored at −80°C. A total of 18 mice were included in the study. The Uppsala Animal Ethics Committee has approved the animal studies (ID number C215/12).

### Ethics Statement

The Uppsala Animal Ethics Committee has approved the animal xenograft studies (ID number C215/12), and all guidelines were followed. The mice were inspected every day and tumor sizes were measured every second to third day. The mice were sacrificed after blood collection by controlled exposure to increasing concentration of CO_2_. After the mice had died cervical dislocation was performed as an extra measure of security. It can therefore not be considered as a standard survival study since the experiment was terminated before any mouse’s health was put in danger. No animal dies as a result of the intervention. According to the research animal permit a mouse has to be euthanized if the subcutaneous tumor reach a size above 10 mm in diameter or if the tumor gets ulcerous. This was never the case in this study. Anesthetics is not common use for subcutaneous tumor cell injection or blood sampling. It was therefore not used.

### Dried Blood Spot Experiments

Blood was sampled by venipuncture and transferred into EDTA sample tubes. Tubes were inverted 10x directly after sampling. Tubes were stored and transported in an upright position in a dark box at RT for 6 h. Tubes were inverted 10x after which 15 µL was added to pre-marked circles on a Whatman DMPK-C card (GE Healthcare). The blood droplets were allowed to hang from the pipette tip and carefully touch the surface of the DMPK-C card for smooth spreading. Immediately after the spotting procedure 1.5 mL of EDTA blood from each sample tube was transferred to 1.5 mL cryo vials and spun 10 min at 2000×g. Supernatants (plasma) were carefully transferred to new cryo vials stored at −20°C. Dried blood spots (DBS)-cards were dried horizontally over night at RT and placed in separate plastic zip-lock bags together with a Minipax adsorbent packet (Sigma-Aldrich) and stored at RT. After 12 d storage, a 1.2 mm Ø disks were punched with a fixed 1.2 mm puncher and ejected into separate wells of a 96-well PCR plate. Each disk contained approximately the equivalent of 0.43 µL blood. Punching of a blank filter disk was made in between to prevent unwanted contamination between samples. For analysis purposes, blank disks were run and used as background.

## Results and Discussion

### Oligonucleotide Redesign to Enable Higher Degree of Multiplexing

PLA, and more recently PEA, were developed as sensitive immunoassays to quantify proteins in small volumes of biological samples [6], [7], [8], and stepwise multiplexed to a level of 24 [4], [9], [10], [11]. As a step towards higher degree of multiplexing the probe oligonucleotides (A and B) were redesigned to contain binding sites for both universal (for preamplification) and specific primers (for qPCR detection), one unique 5-base pair site for pairwise annealing between oligo A and B, and one universal site for molecular beacon binding for detection in qPCR (Figure 1A).

Even in the absence of antigen bringing matching probes together, there will be probes in proximity due to random diffusion in the sample. The ratio between incubation volume and extension mix volume has been maximized in the protocol (4 to 100 µL) in order to minimize the concentration of unbound probes at the time of extension, and thereby reducing the background signal which yields higher sensitivity. A unique annealing site was added to each of the oligonucleotides to avoid non-matching probes to bind each other, and thereby further enforce specificity as well as reducing the number of background events. To test if the unique hybridization site would lower unspecific background, a 96-plex PEA was performed as described below but using mismatched primer pairs in the qPCR readout. None of the mismatched primer pairs gave any detectable qPCR signal, which verified the stringency of the annealing site (Data not shown).

To be able to efficiently screen for new biomarkers, perform protein expression profiling, and combine several biomarkers for increased power of discrimination, high multiplexing level is essential. Subsequent to the development of singleplex and 24-plex PEA [8], we chose to increase the multiplexing to a level of 96, and with a focus on cancer-related biomarkers. A set of 92 cancer-related proteins were selected to form the PEA panel. The analytes chosen included well-known cancer biomarkers (e.g. CA125, CA242, CEACAM5), proteins known to be selectively increased in cancerous tissue (e.g. AM, ErbB2, Fas), inflammatory proteins (e.g. IL-6, IL-8, CD69), proteins involved in angiogenesis (e.g. VEGF, EGF, HGF), as well as several exploratory proteins (Table S1).

For normalization purposes, four internal controls were included: Two incubation controls use PEA probes to quantify the non-human proteins phycoerythrin (PE) and green fluorescent protein (GFP). These were used to for quality control to identify possible outliers amongst the samples, and for evaluation of normalization procedure as their endogenous levels in all samples is known to be zero. The extension control was an antibody linked to both A and B oligonucleotides providing immediate proximity independent of antigen binding. Finally, a detection control made up of a fixed amount of a complete double-stranded template amplicons monitored the variability in the preamplification and qPCR steps. The extension control was found to most efficiently reduce variation across different samples and was therefore chosen as a normalizer in all data analysis herein (see Materials and Methods).

94 pairs of analyte-specific PEA probes were generated (including the two incubation controls), as described above, and combined in pools containing either all of the A or B probes (for the extension control and detection control there were no probes) at 1.33 nM (Table S1; Figure 1A). Multiplexed proximity extension assays where performed by first incubating 1 µL of a sample with 3 µL of the proximity probe mix (containing both A and B probes at 133 pM) at 4°C over-night in a standard PCR-type microtiter plate. This allows for binding of the probes to their respective target analytes (Figure 1B). Next, 96 µL of a proximity extension/preamplification solution containing nucleotides, universal primers, and DNA polymerases was added at RT (described in the paragraph below). The micro titer plate is placed in a PCR thermocycler for the proximity extension to take place at 50°C (Figure 1B) and then immediately goes into a preamplification cycling program using the universal PCR primers amplifying all the 96 different types of amplicons in parallel (Figure 1C). An uracil-DNA-glycosylase (UDG enzyme was added to all samples to digest remaining primers (Figure 1D). Each preamplification reaction is finally transferred to a microfluidic qPCR chip along with the respective target-specific primer pairs and a molecular beacon for fluorescent monitoring and detection in each of the 96 samples across 96 assays simultaneously. Cq-values derived were normalized as described above, and the resulting ddCq-value level is proportional to the initial protein level in the sample (Figure 1E).

### Improved Enzymatic Performance using a Hyper-thermostable DNA Polymerase

PEA was initially developed as an immunoassay performed without washing steps, which increased precision compared to solid-phase assays [8]. However, the T4 DNA polymerase used to unite the two PEA probes and digest unbound probes in the first version of PEA is active at RT. Therefore, the addition of extension/preamplification mix had to be performed in a pre-heated thermocycler to avoid undesired background to form upon the addition of the enzyme. For an assay to be useful in clinical diagnostics, low experimental hands-on time and robustness are important parameters. To meet these requirements, the PEA protocol had to be significantly improved by reducing the number of pipetting and incubation steps.

**Figure 2:**
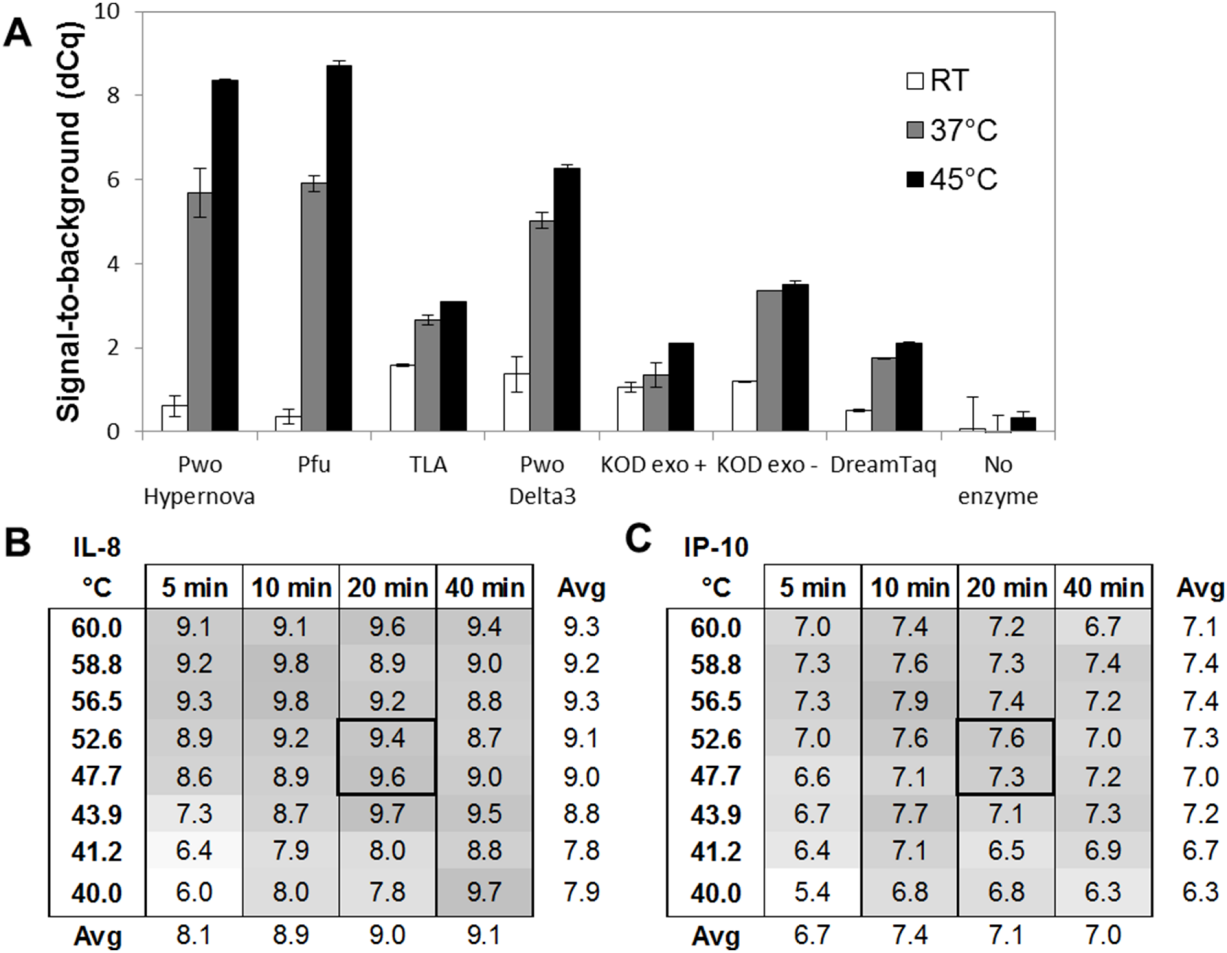
Improved enzymatic performance using a hyper-thermostable DNA polymerase. (A) Seven hyper-thermostable DNA polymerases were compared using an IL-8 assay (10 pM Ag) with regards to their ability to remain inactive during RT addition (low background signal) while efficient at either RT (white bars), 37°C (gray bars), or 45°C (black bars) extension. Pwo Hypernova generated high signal-to-noise values (dCq) and chosen as the most suitable candidate. (B–C) Assessment of optimal time and temperature for the extension reaction using an IL-8 and an IP-10 assay (100 pM Ag). 20 min at about 50°C generated the highest signal-to-noise values (dCq) while retaining robust signals.

We therefore investigated a number of hyper-thermostable DNA polymerases possessing low (or no) activity at RT, which would allow bench-top addition of all reagents at RT, while being sufficiently active at a higher temperature that also maintains the antibody/antigen binding. To do this we tested seven different enzymes (Pwo Hypernova, Pfu, TLA, Pwo Delta3, KOD exo+, KOD exo−, and DreamTaq) with regards to their ability to remain silent during RT addition (low background signal) while being able to efficiently extend at either RT, 37°C, or 45°C (high antigen-dependent signal). PEA probes specific for human IL-8 were used in the analysis, and 10 pM IL-8 was used as antigen. This experiment revealed that three out of the seven enzymes, Pwo hypernova, Pfu, and Pwo Delta3, were the least active during the 10 min RT addition/incubation in the sense that the background in those reactions was similar to that of a reaction without enzyme. These three enzymes performed relatively well at 20 min extension at 37°C, resulting in a signal-to-noise value (dCq) of 4–6 (Figure 2A). When increasing the temperature further to 45°C, both Pwo Hypernova and Pfu gave a higher dCq-value between 8 and 9. Pwo Hypernova was one of the most suitable candidates, and selected for all subsequent experiments.

Next, we pinpointed the optimal time and temperature for the extension reaction to reach the highest possible sensitivity. This was done using a Veriti 96-Well Fast Thermal Cycler (Life Technologies) that is built with independent temperature blocks, and therefore allowed us to evaluate the extension reaction with a temperature gradient. IL-8, IP-10, and CXCL16 assays were used to measure 100 pM of antigen. Extension was performed at eight different temperatures spanning between 40°C and 60°C, and samples were removed from the thermocycler after 5, 10, 20, or 40 min, after which all samples were preamplified and analyzed by qPCR. Figure 2B–C shows the signal-to-noise (dCq) level for each condition for the IL-8 and IP-10 assays, respectively. These data suggested that 20 min extension at a temperature around 50°C was optimal to achieve the highest sensitivity while retaining robust signals in PEA. A CXCL16 assay was also evaluated in parallel and showed similar results (Data not shown).

### Improved Preamplification Protocol Resulting in Increased Precision and Sensitivity

In the initial PEA protocol the DNA polymerase-driven extension uniting the PEA probes and the preamplification were performed in two consecutive steps, including a transfer of sample with a generally low number of templates. Such a transfer is very likely to introduce high stochastic variation. Therefore we merged the two steps by simply mixing the reagents required for both extension and preamplification, and run a combined thermocycler protocol.

As the readout is performed with a microfluidic qPCR system that measures only 7 nL reactions, a preamplification step had to be performed to reach sufficient signal. A rule of thumb, according to Fluidigm’s guide lines, is to use samples with sufficient numbers of templates to get a Cq-value <25 for good precision. We estimated that at least 15 preamplification cycles were required to reach such a high signal. However, a major drawback with using multiple primer pairs in PCR is that there is often a bias between different sequences resulting in improper quantification and poor expression profiling of a set of analyzed biomarkers. In addition, a sample containing very high levels of a certain protein, the corresponding primer pair might be limited and, thus, the signal would be saturated. To circumvent such a bias, and to enable equal amplification of all target sequences, the proximity probe oligonucleotides were redesigned to include binding sites for universal preamplification primers. Also, by replacing two internal tyrosines with 2′-deoxy-Uridine bases, excess primers remaining after preamplification could be efficiently removed with a Uracil-DNA glycosylase (UDG). This removes unspecific background in the qPCR readout. The UDG-driven primer removal was carefully assessed with enzyme titration, and revealed no increase in background due to remaining primers at the concentration chosen (Data not shown). Thorough evaluation of amplification efficiency has been performed on samples with varying protein concentrations and different sequences demonstrating that the present multiplex PCR protocol supported even and efficient amplification above 17 cycles.

**Figure 3:**
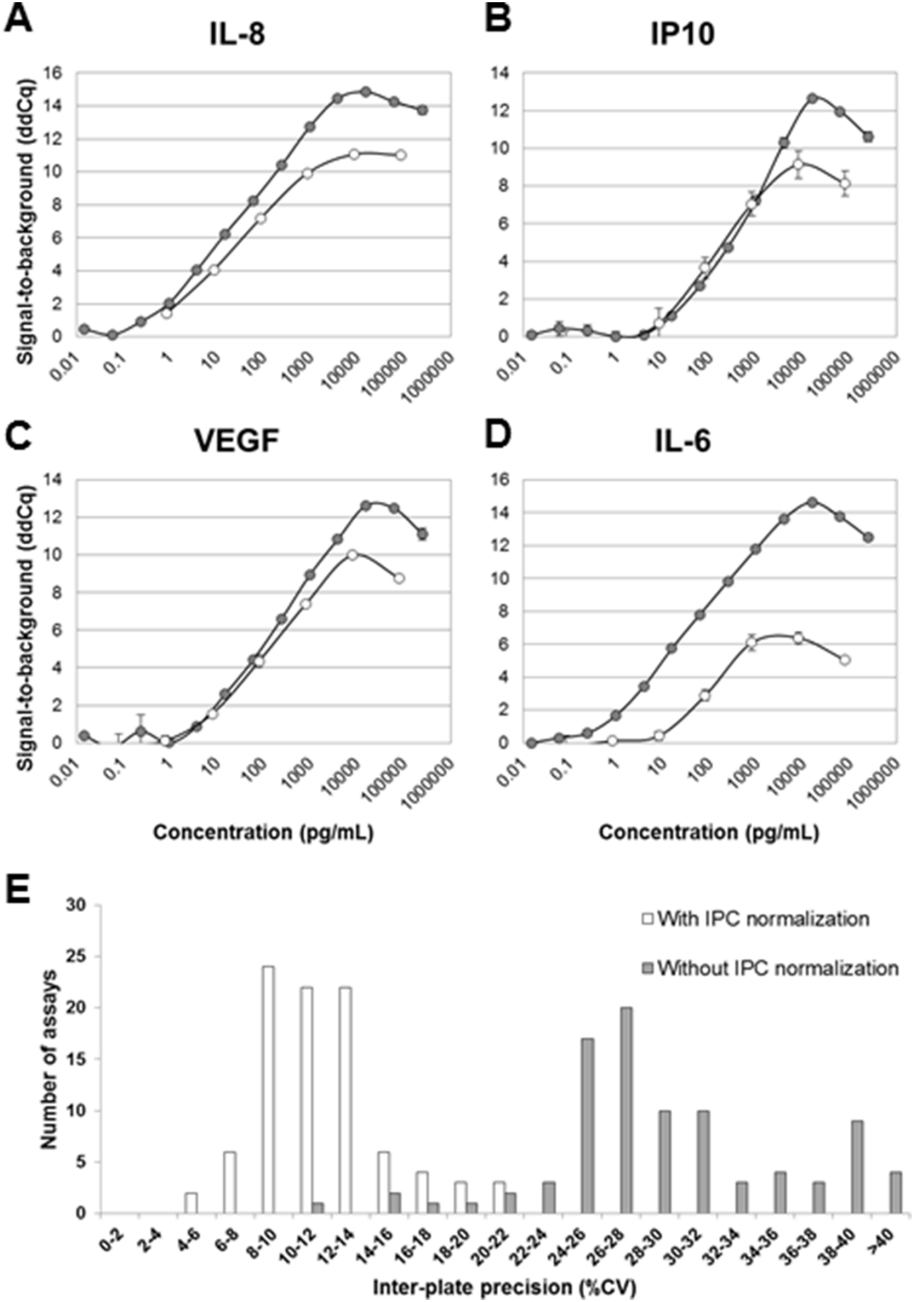
New preamplification and normalization protocols resulting in improved precision and sensitivity. The optimized 96-plex PEA protocol (gray series) was used to analyze standard curves generated for (A) IL-8, (B) IP-10, (C) VEGF, and (D) IL-6 and the results compared that of the initial singleplex PEA protocol (white series) (E) A new normalization procedure was introduced, which significantly reduced the inter-assay precison from average 29 to 12%CV. Histograms show the distribution of CV% across 92 assays and 7 analyzed plasma/serum samples with (white), or without (gray) IPC normalization.

After the optimization work described above, including a new DNA polymerase (Pwo Hypernova), RT addition of extension/preamplification reagents, combined extension/preamplification step, and utilizing universal primers in the preamplification, the PEA protocol was evaluated for four different assays (IL-8, IP-10, VEGF, and IL-6) and compared to data using the old PEA protocol. Standard curves for each of the four assays are plotted in Figure 3A–D. For most assays, there was an improvement of dynamic range and sensitivity as well as intra-assay precision. To evaluate the entire 96-plex panel with regards to critical immunoassay parameters, dilution series of recombinant antigen standards were generated in buffer and analyzed with PEA and a 4-Parameter Logistic (4PL) non-linear regression. Based on this analysis, values for limit of detection (LOD), lower limit of quantification (LLOQ), and upper limit of quantification (ULOQ) for 89 assays were determined (Table S1). Altogether, the sensitivity obtained for the different assays were comparable to the corresponding ELISA from RnD Systems. Roughly half of the assays showed similar, or better, sensitivity (Data not shown). This is quite remarkable considering the fact that no optimizations of individual incubation conditions have been performed in the current PEA panel.

### Inter-Plate Control Improves Precision between Different Runs

The analysis procedure includes a step where each data point is normalized to the extension control signal for the same sample. This improves intra-assay precision by correcting for technical variation. However, this normalization step does not handle variation occurring between different runs. For large studies, including multiple plates and chips, a lower inter-plate variation is desirable. Therefore, we generated a new internal control termed Inter-Plate Control (IPC). This was generated by conjugating goat IgG to each of the 92 different oligonucleotide pairs (similar to the extension control). The 92 different conjugates were individually titrated to generate a Cq-value of around 12, and pooled. A new normalization procedure was evaluated, where the IPC was added as a sample in three positions on each PEA plate, and the median value was calculated for each assay. The median values were finally subtracted from all extension control-normalized dCq-values. A set of plasma and serum samples (n = 6) was analyzed in triplicates with PEA and run on 8 different Fluidigm chips (also different incubations and users), after which inter-plate %CV was calculated with or without IPC normalization. Figure 3E shows that while intra-assay precision remains the same (8%), the average inter-assay precision (reproducibility) improved from 29%CV (gray bars) to 12%CV (white bars) upon IPC-normalization. This demonstrated that with the new IPC normalization one can correct for variation on the assay level and compare data derived from multiple runs. This allows multiplex PEA to be utilized in studies with large sample cohorts. Following these results we decided to introduce the additional step in the normalization procedure, and coined the new term “Normalized Protein Expression (NPX)”, which refers to IPC-normalized data.

### Demonstrating High Specificity and Scalability of PEA

Multiplexing of immunoassays is generally constrained because of false positive signals generated by unspecific binding of antibodies. One of the benefits of PEA and other proximity probing techniques is the high specificity. This is brought about by two things: proximal requirement of the antibodies to generate a signal (similar to sandwich assays), and the use of specific primers in the qPCR detection. As mentioned above, higher multiplexing level is desirable for higher assay throughput in biomarker screenings. The risk of unspecific events in any immunoassay system is exponentially increased with the level of multiplexing. Therefore, we decided to add a third level of specificity to the system when increasing from 24- to 96-plex. This was done by adding a unique 5-base pair long annealing site that prevented non-matching probes to bind as described above.

**Figure 4:**
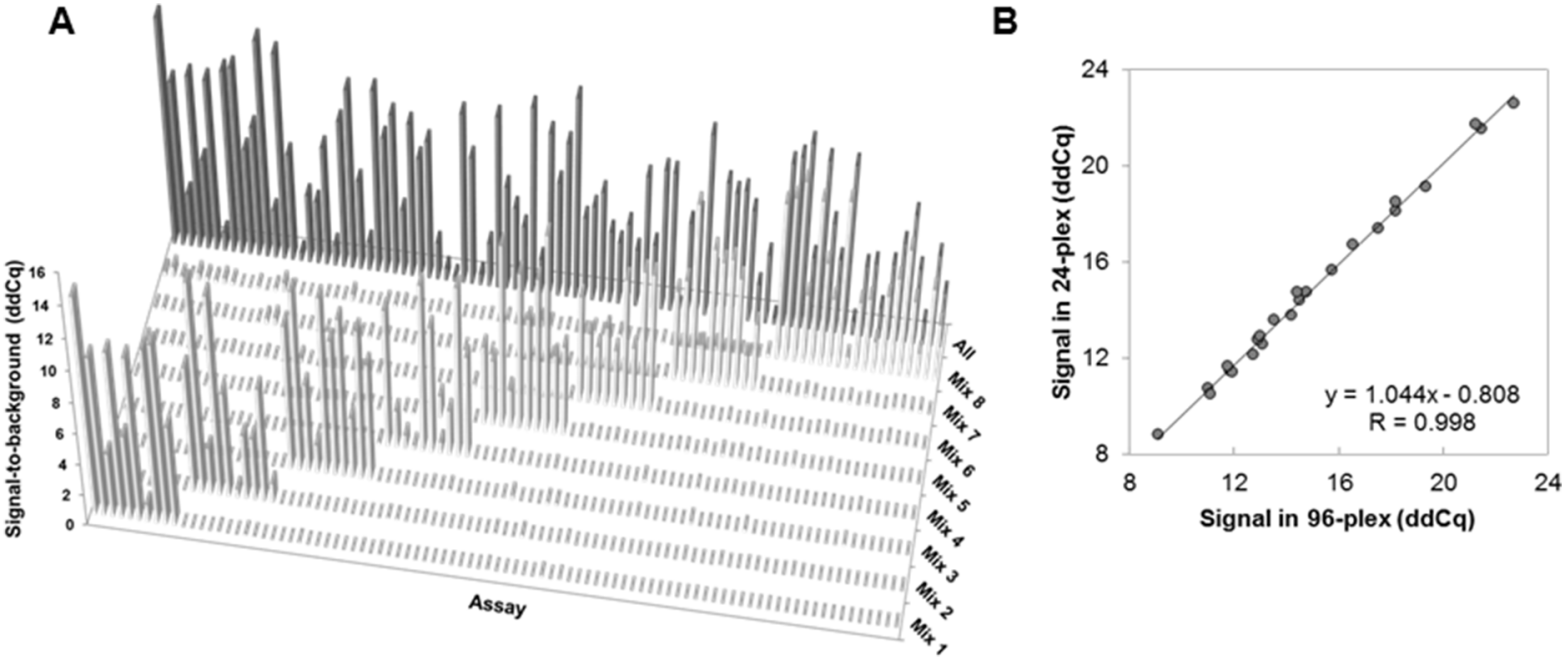
Demonstrating high specificity and scalability of 96-plex PEA. (A) Submixes of antigens were analyzed and demonstrated that the different assays only responded to the submix containing the corresponding antigen and at signal-to-noise levels similar to that of the mix containing all antigens. (B) Scalability was assessed by analyzing 24 PEA assays either in 24-plex or 96-plex, and the normalized levels plotted and compared in an xy scatter. This demonstrated a Pearson correlation value (R) as high as 0.998.

To evaluate the specificity of the PEA, the 90 antigens where divided into eight sub mixes comprising assays (by sequence ID) 1–11, 12–22, 23–32, 33–42, 43–54, 55–64, 65–74, and 75–96, respectively (Table S1). The response for each of the assays was measured either against the different antigen sub mixes (Figure 4A, light gray bars) or against the entire antigen pool (Figure 4A, dark gray bars), all at 5 ng/mL. This showed that for each assay a significant response was only seen with the antigen mix containing the corresponding antigen. This experiment validated the high specificity of the PEA.

Furthermore, the response detected for entire antigen pool was similar to that of the antigen sub mix (Table S1). On average, the difference in ddCq between the mix containing all antigens and each sub mix was as low as 0.1 (equivalent to a 7% change in signal), and with the highest difference observed for adrenomedullin at 0.8. These data demonstrate that the PEA scales well up to 96-plex (Table S1). Scalability was further analyzed by measuring the level of 24 analytes in a healthy plasma sample either in 24-plex or in 96-plex PEA. This was done by generating two different probe mixes. The results are presented in Figure 4B as an xy-scatter plot demonstrating a next to perfect Pearson correlation (R = 0.998; y = 1.044x−0.808) between the two versions of PEA, further supporting the scalability of the system.

As both monoclonal and polyclonal Abs were used to build the multiplex PEA panel it was important to validate that different Ab types would give similar quantifications of an analyte in biological samples. Four different IL-6 assays were generated comprising either mAb/mAb, mAb/pAb, pAb/mAb, or pAb/pAb and used in a side-by-side comparison to measure and quantify IL-6 in three EDTA plasma samples from healthy individuals. A 4-Parameters non-Linear Regression analysis resulted in similar IL-6 concentrations (8–18% CV between the back-calculated concentrations derived from the different assays) regardless of which Ab was used to build the PEA assay (Figure S1 and Table S2). These data further highlighted the high specificity of the multiplex PEA.

The fact that new PEA assays are being developed without individual optimizations and that both pAbs and mAbs can be used to generate probes and used together in the same multiplex protocol makes development both flexible and rapid. As for all immunoassays, antibody affinity is the most important and limiting parameter for successfully setting up new PEA assays, where high affinity provides both better sensitivity and dynamic range [12]. As the multiplex assay includes 92 different antibody pairs of which the majority are polyclonal we anticipate a very broad range of affinities in the multiplex assay. However, the incubation conditions have been optimized to function regardless of the source/type of antibody used and for antibodies with different affinities.

Taken together, the current study demonstrates high specificity and scalability of PEA, and illustrates that the technique is likely to work well even at multiplexing beyond 96-plex.

### Function and Lack of Cross-reactivity in Biological Samples

Normal and pathological human plasma samples contain a number of endogenous interfering substances, such as heterophilic antibodies possessing broad reactivity with antibodies of other animal species, and are therefore likely to hamper the performance of an immunoassay [13]. To evaluate the potential impact of this specific interference, a special “mismatch” system was designed in which the probe oligonucleotide A contained a modified 3′-end to allow annealing with a mis-matched probe oligonucleotide B. The only way to generate a signal here is by mis-matching antibody probe pairs being brought into proximity by cross-binding substances other than antigens, e.g. heterophilic antibodies and similarly acting rheumatoid factor. Six different mis-matched probe pairs of varying antibody host species origins were designed and evaluated with a Heterophilic Assessment Panel from Scantibodies Laboratory Inc. (part no. 3KG027) containing samples with varying confirmed levels of interfering substances. No interference could be detected for any of the panel samples, indicating a sufficient blocking ability during the incubation step (Data not shown).

**Figure 5:**
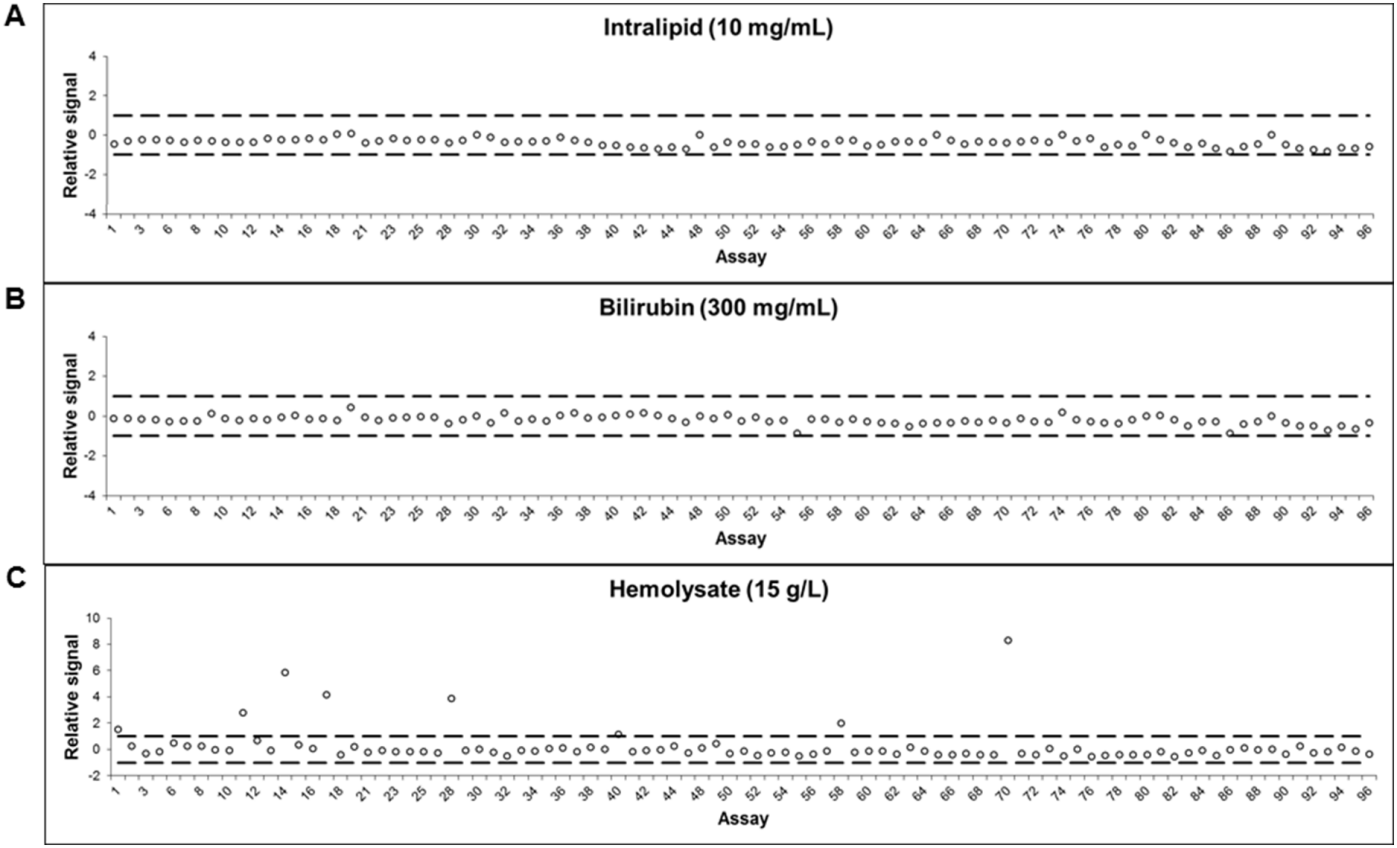
Low interference in biological samples. To determine whether PEA was affected by interference, three known interfering substances were spiked in plasma and measured by 96-plex PEA. No interference was seen with plasma containing (A) bilirubin or (B) intralipid, while a few assays showed increased signal in plasma containing (C) hemolysate, most likely due to actual analytes leaking out from the disrupted blood cells.

Other known serum and plasma components known to affect immunoassay performance are hemolysate, lipids, and bilirubin. Serial dilutions of these components were performed and added to normal serum samples to represent different patient health conditions and/or sample collection irregularities. The potential impact of the different additions was assessed by performing PEA on the modified samples and the results were compared to that of normal sera. No interference was detected by addition of bilirubin or intralipid even at concentrations of 300 µg/mL and 10 mg/mL, respectively (Figure 5A–B). This showed that PEA performs efficiently even in extreme conditions, such as hyperbilirubinemia, percholesterolemia, and hypertriglyceridemia [14], [15]. Furthermore, 7 out of 92 assays displayed a significant increase in signal when adding 15 g/L hemolysate to the serum (Figure 5C). Such a hemolysate level corresponds to complete lysis of 10% of all blood cells in a sample (see Materials and Methods). The increase in signal was observed only for a few assays and is most likely due to actual analytes leaking out from the disrupted blood cells rather than disturbance of the assay mechanism.

### Detection of Human Proteins in Sera from Xenografted Mice and in Human Dried Blood Spots (DBS)

**Figure 6:**
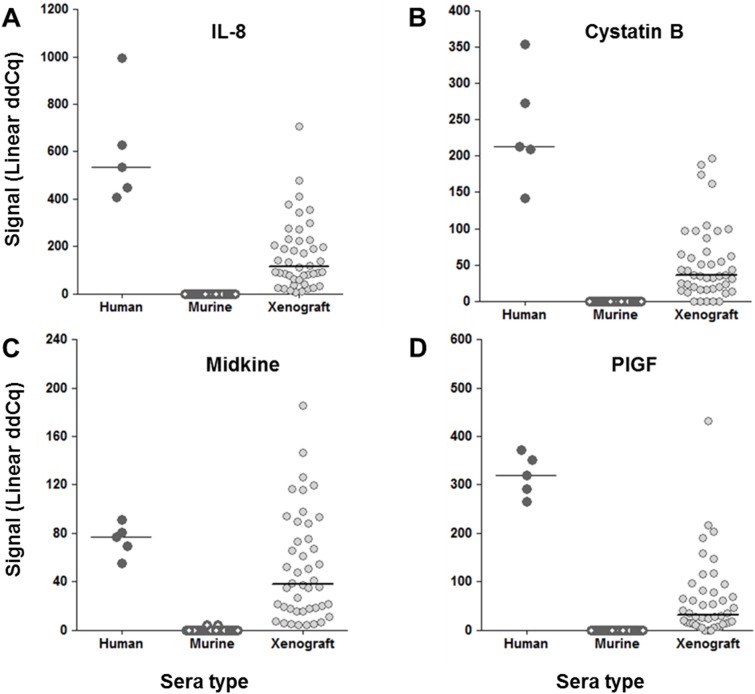
Detection of human proteins in sera of xenografted mice. Nude mice were grafted with human tumor cells (SK-N-FI). Before (white symbols) and on day 30 after inoculation (gray symbols), the sera were analyzed with 96-plex PEA and compared to that of normal human sera (black symbols). Shown are ddCq-values for 4 examples of protein assays: (A) IL-8, (B) Cystatin B, (C) Midkine, and (D) PlGF, for which significant levels of human protein were detected in xenografted mice while being undetected in normal mice.

Low sample consumption is of particular importance for studies using small animal models. As mentioned above, PEA consumes only minute amounts of samples - as little as one µL is enough to detect and quantify 92 different proteins. The nude mouse is an immune-compromised mouse model lacking T lymphocytes and is utilized to study various cancer treatments as it mounts no rejection response towards foreign cells [16]. A xenograft mouse model was established in which female nude mice were grafted with 5×10^6^ SKNFI human neuroblastoma cells subcutaneously in the flank. Three to four weeks after tumor inoculation, mice were bled and sera were prepared. Sera from normal nude mice and xenografted mice were analyzed with PEA. This analysis identified 13 proteins (IL-8, GDF-15, PlGF, E-selectin, Cystatin B, MCP-1, CSF-1, FAS, EMMPRIN, ErbB2/Her2, Midkine, U-PAR, MIC-A, Prostasin) that were detected in xenografted mice while being undetectable in normal mice (Figure 6A–D; Data not shown). The plots present the detected protein levels in xenografted mice with levels in normal human sera as a comparison. This showed that proteins from the grafted human tumor cells efficiently leak out to the circulation of the mouse and can be detected by PEA. Several proteins (n = 30) were also detected in normal mouse sera (Data not shown). This correlated well with reported antibody cross-reactivity between mouse and man in most cases.

Dried blood spot testing (DBS) is a form of biosampling where blood samples are blotted and dried on filter paper [17]. Capillary blood, obtained from pricking the heel or finger and blotted onto filter paper, is routinely used to screen for metabolic diseases in large populations of newborns. The technology holds promise to expand to diagnostic testing in resource-poor and rural areas due to the long sample lifespan and easier transportation independent of refrigeration [18].

**Figure 7:**
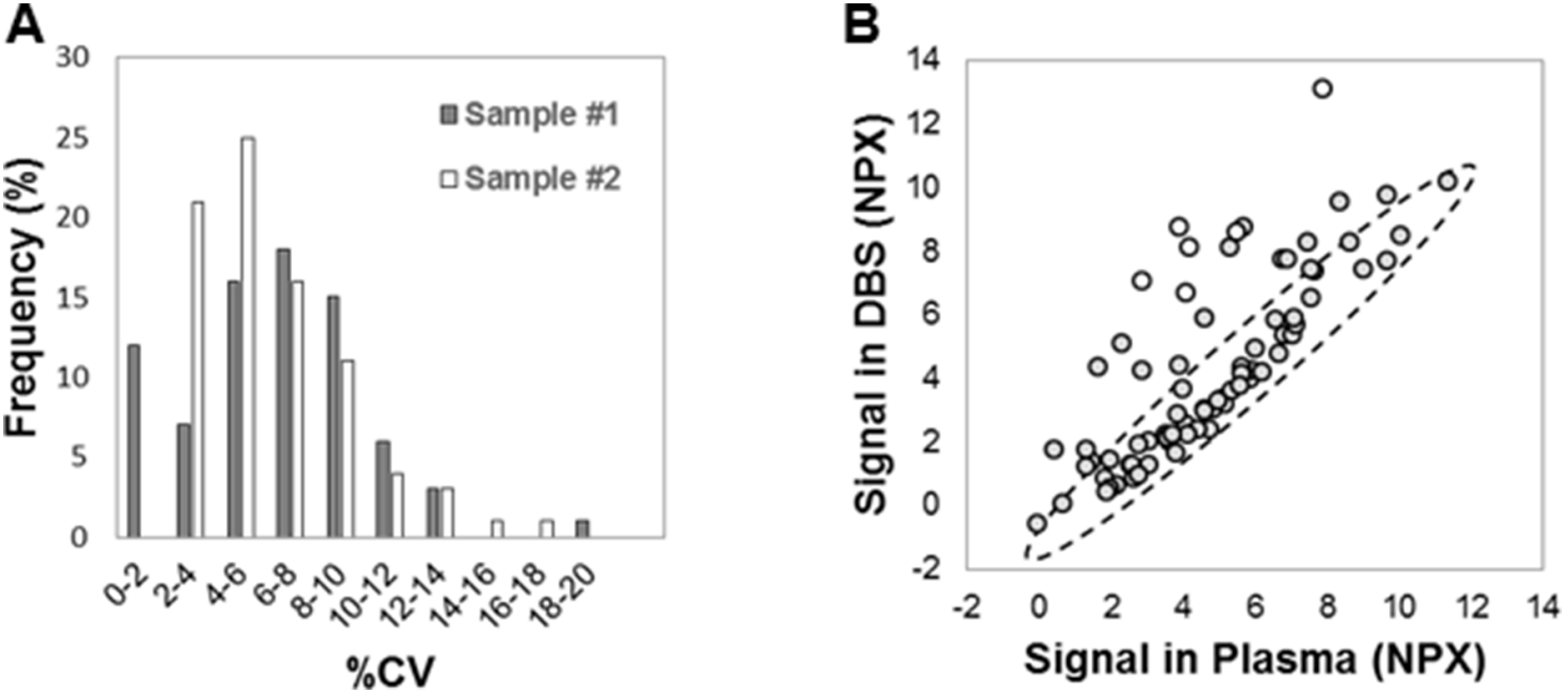
Applying multiplex PEA to the analysis of dried blood spots (DBS). DBS samples were analyzed by 96-plex PEA. (A) Inter-spot precision was assessed in duplicate samples. Shown is the distribution of %CV across all assays for two individuals (gray and white). (B) EDTA DBS and EDTA plasma samples from the same individual were analyzed. The normalized protein expression es (NPX) is shown as an XY scatter plot demonstrating a Pearson correlation value (R) of 0.75 between the two sample types (gray series).

One limitation with DBS samples when it comes to protein analyses, however, is the very low sample volume. As low sample consumption is one of the hallmarks of PEA, we applied the 96-plex PEA to DBS analysis. EDTA plasma and EDTA DBS were prepared in parallel from two healthy donors (see Materials and Methods). From each DBS 1.2 mm Ø disks (spots) were excised and transferred to a tube, after which 1 µL PBS, probes and incubation solution were added, followed by a regular MUX PEA protocol (as described above) with the disk remaining in the tube until the qPCR step. Inter-spot precision (%CV) was determined for duplicate spots, and for each assay. This demonstrated a very high precision with an average of 4.3 and 6.4 CV% across all assays. Figure 7A displays the distribution of precision for the two DBS samples in a histogram. Next, we compared the signal detected for each of the analytes and compared the results to that of EDTA plasma. For some assays there was an increased level of protein detected in EDTA DBS when compared to EDTA plasma (Figure 7B). These included six analytes, IL-8, Cystatin B, Galectin-3, CXCL11, Myeloperoxidase, and Caspase-3 (shown as white circles), whose levels were significantly increased in Hemolysate-containing serum in Figure 5. This experiment revealed a correlation between protein expression levels determined in the two samples types (Pearson correlation = 0.75 calculated on all analytes excluding the six mentioned above). This suggests that for some analytes there is an additional release from the blood cells after addition to the paper, partly because of cell disruption. In line with this, a previous study has demonstrated a correlation between DBS and plasma when measureming therapeutic mAb concentrations in monkeys [19]. The authors also found a two-to-three-fold differences in detected levels, possibly attributable to hematocrit or differences in recovery. All-in all,our data demonstrate that the DBS sample format is highly compatible with multiplex PEA.

DBS sampling requires minimal amount of blood, and is therefore particularly well suited for infant screening by capillary heel stick. A well working method to examine blood from extremely premature infants could give unique information on health related issues, which hopefully could result in increased premature infant survival in the future. Such analysis relies on accurate and highly multiplexed analyses on very limited sample amounts, which makes the 96-plex PEA assay a possible candidate for this demanding task. Besides minimal patient invasiveness, DBS sampling is also very simple in terms of storage and transportation. This makes DBS sampling well suited for home testing for e.g. long-term follow up after treatment or health status controls were the individual handles the sampling and sends the DBS by regular mail for analysis and medical evaluation. Another aspect to the DBS ease of sampling, storage and transportation are screening for certain diseases, such as tuberculosis, that are common in developing countries where resources and infrastructure are limited. The PEA technology is a suitable way to perform quantitative analysis of protein markers from DBS samples, and could possibly be applied to all areas discussed above.

Altogether, the DBS and xenograft studies both demonstrate a good performance of multiplex PEA in biological samples. More importantly, these are two applications that are compatible with multiplex PEA when low sample consumption is critical.

## Concluding Remarks

Rapid and scalable immunoassay development is desired to increase knowledge of disease mechanisms and drug activity, and holds promise for early disease detection [20]. The presented PEA platform can be scaled to detect 92 proteins in 96 samples simultaneously, opening up new opportunities for large-scale data generation of protein biomarkers. PEA encompasses high specificity, high sensitivity, and low sample consumption - three important immunoassay parameters. The minute sample consumption makes PEA particularly valuable for studies using scarce samples, such as fine-needle biopsies, cell lysates from precious cells, and small model animals. Single-cell research is accumulating growing evidence of importance for understanding cancer development, future drug development and for diagnostics aiming to provide the right treatment to the responsive fraction of the patient population [12]. Recently a nucleic acid proximity assay was shown to quantify proteins even in isolated single cells [21]. As the homogeneous PEA can scale in volume due to the absence of solid support and have strong amplification of signal through PCR, it is especially suited for large-scale single-cell analyses.

We envision the development of disease area-focused panels of protein biomarkers with the thousands of commercially available antibodies to be suitable for this purpose. As the specificity of multiplex PEA is controlled at several levels, and the PEA is shown to be scalable, we foresee no problems to further increasing the level of multiplexing.

## Supporting Information

Figure S1 **IL-6 assays made up of either mAb, pAb, or a mix give comparable determinations of protein concentration.** Four different IL-6 PEA assays were generated that were made up of either mAbs, pAbs, or a mix. Antigen standard curves were generated with recombinant human IL-6 and used to quantify IL-6 in three different EDTA plasma samples (green triangle, purple square, red circle).
(TIF)

Table S1 Panel description, sensitivity parameters, and specificity data.
(XLSX)

Table S2 IL-6 assays made up of either mAb, pAb, or a mix give comparable determinations of protein concentration. Four different IL-6 PEA assays were generated that were made up of either mAbs, pAbs, or a mix. Antigen standard curves were generated with recombinant human IL-6 and used to quantify IL-6 in three different EDTA plasma samples. A 4-Parameter Logistic (4PL) non-linear regression analysis was performed and the IL-6 levels were determined in pg/mL for each sample.
(XLSX)
