# Supplementary figures and images for: Homogenous 96-Plex PEA Immunoassay Exhibiting High Sensitivity, Specificity, and Excellent Scalability
Source: PLoS One. 2014 Apr 22;9(4):e95192. doi: 10.1371/journal.pone.0095192 (PMC3995906; doi:10.1371/journal.pone.0095192)

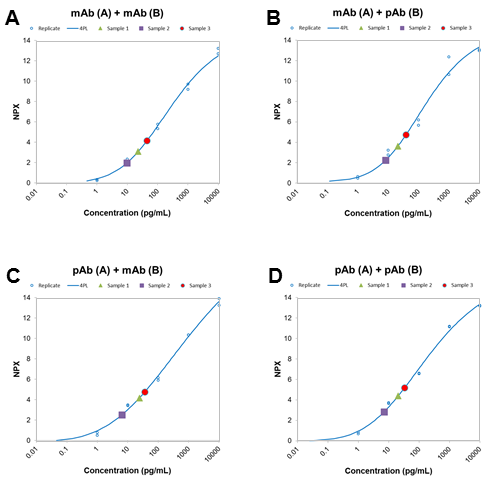

Supplement: Figure S1 — IL-6 assays made up of either mAb, pAb, or a mix give comparable determinations of protein concentration. Four different IL-6 PEA assays were generated that were made up of either mAbs, pAbs, or a mix. Antigen standard curves were generated with recombinant human IL-6 and used to quantify IL-6 in three different EDTA plasma samples (green triangle, purple square, red circle). (TIF) [file pone.0095192.s001.tif]
